# Does Dual Eligibility Drive Health Care Use Patterns? Evidence From MEPS Using Latent Class Analysis

**DOI:** 10.1093/geroni/igaf122.388

**Published:** 2025-12-31

**Authors:** Mohammad Usama Toseef, Ramin Homayouni, Aastha Dharia, Wassim Tarraf

**Affiliations:** Corewell Health, Farmington Hills, Michigan, United States; Oakland University, Rochester, Michigan, United States; Oakland University William Beaumont School of Medicine, Rochester Hills, Michigan, United States; Wayne State University, Detroit, Michigan, United States

## Abstract

Dually-eligible beneficiaries (or duals) are enrolled in both Medicare and Medicaid. Duals have high and complex healthcare needs, and face substantial barriers to quality healthcare access. Thus, it is important to understand how dual-eligibility status is associated with patterns of healthcare services use and the factors that drive these patterns. We used data from the Medical Expenditure Panel Survey (2008-2022) to evaluate the association of dual-eligibility status and health care use patterns for older Medicare beneficiaries (65+-years). We used Latent Class Analysis to sequentially fit and assess multiple class solutions based on 10 healthcare services (e.g. hospital use, dental visits, home healthcare, medications) and generated healthcare use patterns profiles. Subsequently, we fit survey-weighted unadjusted and adjusted multinomial logistic regression models to link dual-eligibility status to the best fitting use patterns profiles. We found that the three class solution (typical [67.8%], high [17.5%], and low [14.7%] users) provided the best statistical and substantive fit to the data. Unadjusted models showed that duals are more likely to be both high (RRR = 2.07, p < 0.001) and low users (RRR=1.45, p < 0.001) as compared to non-duals. After adjusting for sociodemographics, comorbidities, and access to usual source of care, duals remained more likely to be high users (RRR=1.7, p < 0.001), but no longer low users as compared to non-duals. These results highlight the need for more nuanced approaches, by stakeholders, to policies and interventions that streamline the use of health services in a way that aligns with efficient, quality and cost-effective healthcare for this complex need population.

